# Three-dimensional Stereoscopic Visualization Shortens Operative Time in Laparoscopic Gastrectomy for Gastric Cancer

**DOI:** 10.1038/s41598-019-40269-3

**Published:** 2019-03-11

**Authors:** Yoshiro Itatani, Kazutaka Obama, Tatsuto Nishigori, Riki Ganeko, Shigeru Tsunoda, Hisahiro Hosogi, Shigeo Hisamori, Kyoichi Hashimoto, Yoshiharu Sakai

**Affiliations:** 1 Department of Surgery, Graduate School of Medicine, Kyoto University, Kyoto, 606-8507 Japan; 2 Department of Surgery, Kyoto City Hospital, Kyoto, 604-8845 Japan

## Abstract

Laparoscopic gastrectomy for gastric cancer is now widely accepted and has become a standard surgery. This study investigated the advantages of three-dimensional (3D) stereoscopic visualization for laparoscopic gastrectomy over a conventional two-dimensional (2D) planar screen. The primary outcome of this study was operative time. Ninety-four consecutive cases of gastric cancer patients who underwent laparoscopic total gastrectomy (LTG) (25 cases) or laparoscopic distal gastrectomy (LDG) (69 cases) were enrolled in this study before and after the introduction of the 3D system. Operative time was significantly shorter in the 3D groups for both LTG (351 vs. 406 min, *P* = 0.026) and LDG (269 vs. 344 min, *P* < 0.01). During intracorporeal procedures, dissection time was significantly shorter in the 3D groups for both LTG (183 vs. 232 min, *P* = 0.011) and LDG (161 vs. 213 min, *P* < 0.01), although the time needed for anastomosis was similar between the groups. However, operators preferred intracorporeal knot-tying as a ligature for anastomosis under 3D (LTG, *P* = 0.012; LDG, *P* < 0.01). These data suggest that 3D stereoscopic visualization shortens the operative time of laparoscopic gastrectomy by reducing the intracorporeal dissection time.

## Introduction

Laparoscopic gastrectomy has been widely accepted as a minimally invasive surgery and has become one of the standard surgeries for early gastric cancer. Although there is no evidence for the oncological superiority of laparoscopic surgery over open surgery for early gastric cancers, recent meta-analyses have demonstrated its clinical benefits such as fewer postoperative complications and shorter postoperative hospital stays^1,2^. Moreover, its application has been extended to advanced gastric cancers that require D2 lymphadenectomy and/or combined resections^3,4^. However, it can still be technically challenging, especially when advanced lymphadenectomy or oesophagojejunostomy (E-J) is required. For these advanced procedures, the magnifying effect of the laparoscope is an advantage. Conversely, the lack of depth perception with a conventional two-dimensional (2D) laparoscopic planar screen may be a disadvantage that frustrates surgeons, resulting in increased operative time.

Recent advances in surgical devices are outstanding with new, innovative designs available almost every year. Three-dimensional (3D) stereoscopic laparoscopy is one such recent innovation. To date, there are some reports showing the benefit of 3D stereoscopic visualization in dry box training for tasks that require high levels of precise spatial perception, such as suturing and knot-tying^5,6^. In addition to the benefit of dry box training, there are some reports showing the clinical benefit of 3D displays during laparoscopic/thoracoscopic surgeries in urology, gynaecology, thoracic and rectal surgery^7–11^. As for laparoscopic gastric surgery, there were no previous studies showing the direct benefit of 3D stereoscopic visualization to reduce total operative time, although some have shown only a limited 3D benefit^12,13^. Therefore, a detailed study was necessary to draw conclusions about the possible benefit of 3D stereoscopic visualization for laparoscopic gastrectomy for gastric cancer.

Here, we report that 3D stereoscopic visualization shortened total operative time for both laparoscopic total gastrectomies (LTG) and laparoscopic distal gastrectomies (LDG) for gastric cancer. We set operative time as the primary outcome and compared 94 consecutive cases of gastric cancer patients who underwent LTG or LDG before and after introduction of the 3D system at our hospital. Multivariate analyses revealed that operative time performed under 3D was significantly shorter than under 2D. We measured time needed for intracorporeal dissection and anastomosis and found that dissection time under 3D was significantly shorter, although anastomosis times were similar. Our data demonstrates the benefit of 3D stereoscopic visualization during laparoscopic gastrectomy for gastric cancer by confirming shorter operative times.

## Methods

### Patients

We started using the Olympus 3D laparoscopic system (LTF-190-10-3D ENDOEYE FLEX 3D) in May 2017, at Kyoto University hospital. A total of 94 consecutive patients (25 LTG and 69 LDG) who underwent LTG or LDG for primary gastric cancer 12 months before and after introduction of the 3D system were evaluated in this study. Patients who underwent open surgery, robot-assisted surgery, laparoscopic proximal gastrectomy, remnant total gastrectomy or additional splenectomy were excluded. Of these 94 patients, 13 LTG and 40 LDG patients underwent surgery using the Olympus 2D flexible scope (LTF-S190-10 ENDOEYE) with planar monitors, whereas 12 LTG and 29 LDG patients underwent surgery using the Olympus 3D flexible scope with stereoscopic monitors through polarized glasses. Clinical, surgical, and pathological outcomes of the patients were retrospectively analysed. The study protocol was approved by the institutional review board of Kyoto University (approval number R1537) and was performed in accordance with its guidelines and regulations. All patients provided written informed consent for the use of their clinical data.

### Surgical procedures

**Figure 1:**
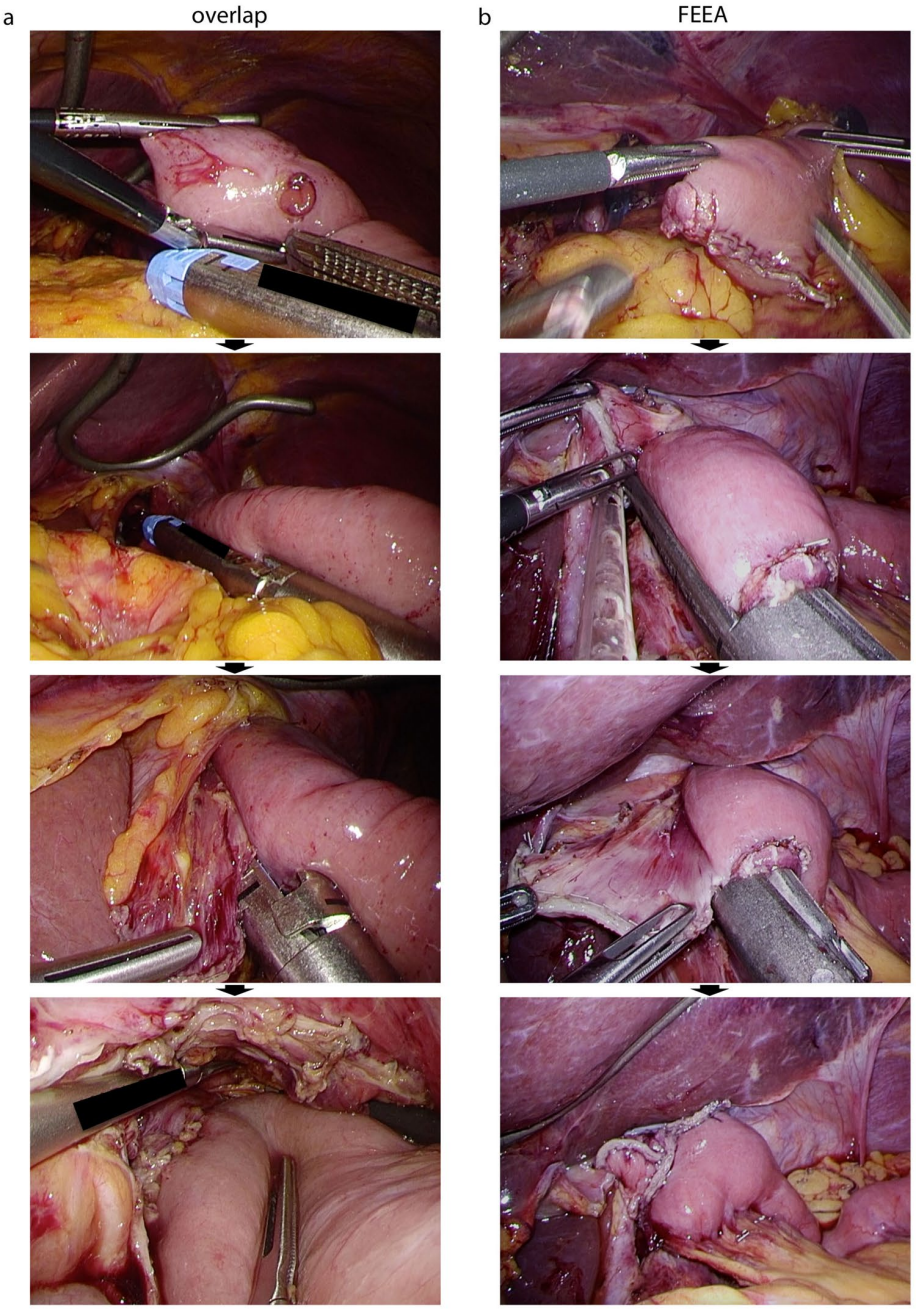
Representative pictures of two types of E-J anastomosis for LTG. (**a**) overlap method. After creating two holes, one on the oesophagus stump and the other on 5–7 cm distal from the jejunum stump (top panel), side-to-side anastomosis is constructed with a linear stapler (2^nd^ and 3^rd^ panel). Then the entry hole is closed (bottom panel). (**b**) FEEA. After creating two holes on the oesophagus and jejunum edge (top panel), a linear stapler is inserted to construct an anastomosis (2^nd^ and 3^rd^ panel). Then the entry hole is closed (bottom panel).

**Figure 2:**
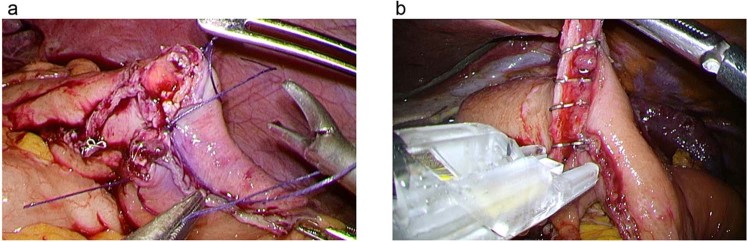
Representative pictures of intracorporeal anastomosis (gastrojejunostomy of Roux-Y reconstruction). After creating an anastomosis hole between the oral and anal sides with a linear stapler, the entry hole of the first liner stapler was temporarily closed using IKT (**a**), LHS (**b**), EKT or running suture. The hole was then completely closed using an additional linear stapler.

All surgeries were performed under the supervision of one of four qualified endoscopic surgeons (KO, ST, HH, or SH) who are board certified by the Japan Society of Endoscopic Surgery^14^. Over the last 10 years at our institute, we have performed 69–107 annual gastric cancer operations (Supplementary Figure S1). The level of lymphadenectomy, either D1 + for early gastric cancer or D2 for advanced cancers, was determined by a pre-operative clinical stage evaluation, based on an upper gastrointestinal endoscopy, an upper gastrointestinal series, and thoracic-abdominal computed tomography in accordance with Japanese gastric cancer treatment guidelines^15,16^. Precise procedures of D2 lymphadenectomy for LTG and LDG are reported elsewhere^17,18^. Roux-Y reconstruction was employed for LTG with intracorporeal E-J and extracorporeal jejunojejunostomy (J-J). For E-J, linear stapled anastomosis was performed by either the overlap method or the functional-end-to-end anastomosis (FEEA), based on the cancer location^19^. For tumours located close to the oesophagogastric junction (EGJ), the abdominal oesophagus must be resected to maintain a proximal surgical safety margin. In these cases, the overlap method was performed^20^. When the tumour was further away from the EGJ, FEEA was performed. The E-J anastomosis procedure is described in Fig. 1 and elsewhere^19^. FEEA was also employed for J-J anastomoses. Petersen’s defect was sutured continuously and intracorporeally, whereas the J-J anastomosis gap was sutured extracorporeally. For LDG, a Roux-Y, Billroth-I, or Billroth-II anastomosis was performed based on previous reports^21,22^. Intracorporeal anastomosis requires two linear stapler cartridges in addition to gut transections, as previously reported^21,23^: one linear stapler is used to create an anastomosis opening with the oral and anal sides of the gut, and the other is used to close the first entry hole. Just before using the second linear stapler, the entry hole of the first stapler has to be closed temporarily either with minimal suturing or laparoscopic hernia staplers (LHS) (Covidien, Dublin, Ireland) (Fig. 2). The procedure used to close the opening [intracorporeal knot-tying (IKT), extracorporeal knot-tying (EKT), LHS or running suture] was determined based on the operator’s preference.

Operative time was defined as the period from skin incision to skin closure. Dissection time was from the first cut of the omentum to the end of the final cut of the stomach for resection. Anastomosis time began at the opening of the first hole and ended with the final staple or suture for anastomosis. For dissection and anastomosis times, we excluded the times when the scope was outside of the abdomen, e.g., during cleaning of the scope lens.

### Postoperative complications

Postoperative complications were defined as greater than Grade 2 of the Clavien-Dindo classification^24^.

### Statistical analysis

All continuous variables are expressed as medians (interquartile range) and were analysed using the Mann-Whitney *U* test. Categorical independence was analysed using Fisher’s exact test. Multivariate analyses were performed with factors that showed associations with *P* values < 0.2 in the univariate analyses. Statistical analysis was performed using JMP software, ver.8 (SAS Institute, Japan).

## Results

### Patient characteristics, operative factors and pathological factors

LTG was performed on 12 and 13 patients in the 3D and 2D groups, and LDG was done on 29 and 40 patients in the 3D and 2D groups, respectively, for one year before and after introduction of the 3D system at our hospital. As shown in Table 1, there were no significant differences between the 3D vs. 2D groups for LTG and LDG with regard to age [LTG, 73 (66–80) years in 3D vs. 66 (64–69) years in 2D, *P* = 0.26; LDG, 70 (63–76) vs. 68 (63–74), *P* = 0.67], sex distribution (LTG, *P* = 0.23; LDG, *P* = 0.61) and body mass index (BMI; <25/≥25 kg/m^2^) (LTG, *P* = 1.00; LDG, *P* = 0.58).

**Table 1:** Patient characteristics.

	LTG			LDG		
	3D	2D	*P*	3D	2D	*P*
	(n = 12)	(n = 13)		(n = 29)	(n = 40)	
Age (years)	73 (66–80)	66 (64–69)	0.26*	70 (63–76)	68 (63–74)	0.67^*^
Sex (M/F)	6/6	10/3	0.23**	20/9	24/16	0.61^**^
BMI (<25/≥25 kg/m^2^)	10/2	10/3	1.00**	20/9	31/9	0.58^**^
OwBC/OwoBC^$^	10/2	8/5	0.38**	14/15	18/22	0.81^**^
T factor (1/2/3/4)	2/1/5/4	7/0/5/1	0.13**	16/3/9/2	19/6/9/6	0.72^**^
Lymphadenectomy (D1 + /D2)	5/7	9/4	0.24**	18/11	19/21	0.33^**^
Anastomosis^$$^ (overlap/FEEA)	1/11	2/11	1.00**			
(R-Y/B-I/B-II)				12/13/4	17/18/5	1.00^**^

^*^Mann-Whitney *U* test, **Fisher’s exact test, ^$^operators with/without board certification, ^$$^FEEA: functional end-to-end anastomosis; R-Y: Roux-Y; B-I: Billroth-I; B-II: Billroth-II.

These were also no differences between the 3D vs. 2D groups for LTG and LDG with regard to the following surgical factors: operator with/without board certification (OwBC/OwoBC) (LTG, *P* = 0.38; LDG, *P* = 0.81), level of lymphadenectomy (D1 + /D2) (LTG, *P* = 0.24; LDG, *P* = 0.33) and anastomosis method (overlap/FEEA in LTG, *P* = 0.59; Roux-Y/Billroth-I/Billroth-II in LDG, *P* = 0.99). All laparoscopic gastrectomies were completed without conversion to open surgery. Blood loss in LDG performed under 3D was significantly less than under 2D [0 (0–0) mL in 3D vs. 3 (0–40) mL in 2D, *P* = 0.010], although it was not different in LTG [12 (0–85) mL in 3D vs. 40 (0–55) mL, *P* = 0.71] (Table 2).

**Table 2:** Operative outcomes.

	LTG			LDG		
	3D	2D	*P*	3D	2D	*P*
	(n = 12)	(n = 13)		(n = 29)	(n = 40)	
Operative time (min)	351 (335–380)	406 (367–465)	0.026^*^	269 (243–326)	344 (288–402)	<0.01^*^
Dissection time (min)	183 (162–203)	232 (190–234)	0.011^*^	161 (128–196)	213 (178–258)	<0.01^*^
Anastomosis time (min)	21 (17–24)	24 (21–31)	0.12^*^	18 (15–21)	19 (16–27)	0.12^*^
IKT/EKT/LHS/running^$$$^	9/2/1/0	2/5/2/4	0.012^**^	26/2/1/0	15/2/22/1	<0.01^**^
Blood loss (mL)	12 (0–85)	40 (0–55)	0.71^*^	0 (0–0)	3 (0–40)	0.010^*^
Dissected lymph nodes	49 (45–59)	48 (41–56)	0.35^*^	41 (29–47)	38 (30–49)	0.77^*^
Metastatic lymph nodes	0 (0–1.5)	0 (0–4.5)	0.85^*^	0 (0–2)	0 (0–2)	0.96^*^
Complication grade ≥2	1 (8.3%)	4 (31%)	0.14^**^	2 (6.9%)	6 (15%)	0.30^**^
Postoperative hospital stay	13 (11–14)	17 (13–23)	<0.01^*^	13 (11–14)	14 (12–19)	0.035^*^

*Mann-Whitney *U* test, **Fisher’s exact test, ^$$$^IKT/EKT: intracorporeal/extracorporeal knot-tying; LHS: laparoscopic hernia stapler; running: running suture

The following pathological findings did not differ significantly between the groups; T factors (LTG, *P* = 0.13; LDG, *P* = 0.72), number of dissected lymph nodes [LTG, 49 (45–59) in 3D vs. 48 (41–56) in 2D, *P* = 0.35; LDG, 41 (29–47) vs. 38 (30–49), *P* = 0.77] and number of metastatic lymph nodes [LTG, 0 (0–1.5) in 3D vs. 0 (0–4.5) in 2D, *P* = 0.85; LDG, 0 (0–2) vs. 0 (0–2), *P* = 0.96] (Table 2).

### Operative time and associated factors

Operative time was significantly shorter in both LTG and LDG when done under 3D stereoscopic visualization compared with 2D visualization [LTG, 351(335–380) min in 3D vs. 406 (367–465) min in 2D, *P* = 0.026; LDG, 269 (243–326) min vs. 344 (288–402) min, *P* < 0.01] (Table 2). It is conceivable that the shorter operative time under 3D is the result of some shorter parts of the intracorporeal procedure compared to those under 2D. To address this possibility, we defined two laparoscopic procedures, intracorporeal dissection and anastomosis, and determined the length of time required for each procedure. Advanced intracorporeal lymph node dissection is one of the most difficult laparoscopic surgery procedures and the time required for intracorporeal dissection was significantly shorter under 3D in both LTG [183 (162–203) min in 3D vs. 232 (190–234) min in 2D, *P* = 0.011] and LDG [161 (128–196) min vs. 213 (178–258) min, *P* < 0.01]. The time required for intracorporeal anastomosis, which is also a difficult procedure during laparoscopic gastrectomy, did not differ significantly between the groups [LTG, 21 (17–24) min in 3D vs. 24 (21–31) min in 2D, *P* = 0.12; LDG, 18 (15–21) min vs. 19 (16–27) min, *P* = 0.12].

Furthermore, operators significantly preferred performing IKT when closing the anastomosis hole during intracorporeal anastomosis in both LTG and LDG (LTG, *P* = 0.012; LDG, *P* < 0.01) under 3D visualization (Table 2), whereas they preferred EKT or LHS under 2D. IKT requires higher levels of spatial perception than does EKT or LHS. Therefore, these data suggest that when using 3D visualization operators finished intracorporeal dissection within a shorter time frame and felt less stressed to perform IKT during intracorporeal anastomosis, which was usually the final step in the operation.

**Figure 3:**
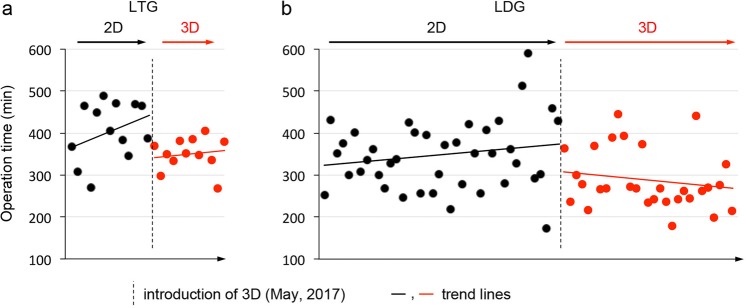
Chronological changes for operative time in LTG (**a**) and LDG (**b**). The X-axis shows the chronological order of the operations. Dotted line denotes introduction of the 3D system (May 2017). Black dots denote operations done under 2D visualization; red dots denote operations done under 3D visualization.

Given that surgeons repeatedly perform the same operation with the same team members, it is conceivable that operative times become shorter because of the experience level of the surgeon and/or the surgical team. In order to assess whether the shorter operative time with 3D visualization was purely the result of surgical team experience, we drew chronological trend lines for operative times before and after introduction of the 3D system (Fig. 3). These did not show clear chronological improvement, but rather clear gaps before and after introduction of the 3D system. This suggests that shorter operative times resulted from the introduction of 3D laparoscopic system.

Next, we compared operative times according to several factors: type of visualization (3D vs. 2D), reconstruction method (overlap vs. FEEA in LTG, and Roux-Y vs. Billroth-I vs. Billroth-II in LDG), World Health Organization T factors (T1 vs. ≥T2), level of lymphadenectomy (D1 + vs. D2), operators (OwBC vs. OwoBC), BMI (<25 vs. ≥25 kg/m^2^) and sex (female vs. male) (Table 3). In the LTG group, univariate analyses revealed that the type of visualization and BMI were factors significantly associated with operative time. In the LDG group, the type of visualization, the reconstruction method, and the level of lymphadenectomy were significantly associated with operative time. We performed multivariate analyses with factors that showed associations with *P* values < 0.2 in the univariate analysis, with similar results. These analyses showed that the type of visualization (3D/2D) correlated with operative time for both LTG and LDG, and that 3D stereoscopic visualization corresponded to significantly shorter operative time.

**Table 3:** Factors associated with operative time (min).

		LTG				LDG			
			Univariate	Multivariate	*P*		Univariate	Multivariate	*P*
			*P*	RC*			*P*	RC*	
Type of visualization	*3D*	351 (335–381)	0.026	−58 (−103 to −14)	0.013	269 (243–326)	<0.01	−50 (−79 to −20)	<0.01
	*2D*	406 (367–465)		reference		344 (288–402)		reference	
Reconstruction	Overlap	448 (351–471)	0.21						
	FEEA	375 (336–405)							
	Roux-Y					364 (302–421)	<0.01	81 (49–113)	<0.01
	Billroth-I					269 (244–300)		reference	
	Billroth-II					351 (269–390)		90 (45–136)	<0.01
T factor	T1	369 (345–384)	0.82			299 (253–351)	0.077	reference	0.62
	>T2	383 (342–427)				327 (269–408)		8.3 (−25–41)	
Lymphadenectomy	D1+	358 (308–448)	0.19	reference	0.036	276 (253–335)	<0.01	reference	<0.01
	D2	385 (351–406)		47 (3.4–91)		367 (275–402)		55 (22–87)	
Operators	OwBC	375 (348–405)	0.76			327 (257–392)	0.82		
	OwoBC	384 (308–469)				300 (262–364)			
BMI	<25	359 (335–397)	0.035	reference	0.080	289 (267–351)	0.71		
	≥25	405 (385–469)		46 (−6.7–98)		326 (253–390)			
Sex	Female	369 (336–381)	0.17	reference	0.24	299 (247–393)	0.72		
	Male	387 (347–469)		27 (−19–72)		302 (268–374)			

*RC, regression coefficient.

### Short-term outcomes

In our study, we also assessed whether laparoscopic gastrectomy with 3D stereoscopic visualization affected postoperative short-term outcomes of the patients. There was no difference in the incidence of postoperative complications greater than Grade 2 of the Clavien-Dindo classification between the groups (LTG, *P* = 0.14; LDG, *P* = 0.30) (Table 2 and Supplementary Table S1). However, the postoperative hospital stay of the 3D patients was significantly shorter in both LTG (*P* < 0.01) and LDG (*P* = 0.035).

## Discussion

This is the first study to show a direct benefit of 3D stereoscopic visualization over 2D for laparoscopic gastrectomy with advanced lymphadenectomy and intracorporeal anastomosis. Namely, we found that the total operative time was significantly shorter with 3D than 2D visualization, and that the shorter operative time was not the result of surgical team experience, based on drawing and comparing the chronological operative time trend lines. We tried to identify the exact reason for the shorter operative times and found that intracorporeal dissection under 3D required shorter times than 2D in both LTG and LDG. Intracorporeal anastomosis is usually the final step after a long procedure of lymph node and stomach dissection during laparoscopic gastrectomy. There was no significant difference in anastomosis time between the 3D and 2D groups in LTG and LDG. However, operators significantly preferred IKT during anastomosis under 3D visualization, suggesting that they were less stressed during dissection using the 3D system and chose IKT, which required more advanced skills than EKT or LHS, to close the anastomosis holes. Conversely, when using 2D visualization, they tended to use EKT in LTG or LHS in LDG. This may have been the reason for the lack of difference in anastomosis times between the groups. For example, the use of a LHS can reduce the time required to close a linear stapler entry hole.

Generally speaking, operators tend to choose the easiest anastomosis procedure since they may be tired or feel stressed after the lengthy intracorporeal dissection even if it is more expensive such as using LHS. Therefore, decreased use of LHS and shorter operative times with the 3D system may contribute to more cost-effective operations. The objective finding that operators performed more IKT when using the 3D system compared with the 2D system suggests that they were less stressed throughout dissection under 3D. However, the limitation of this study was that we were not able to assess subjective markers of the surgeons’ stress level, such as State-Trait Anxiety Inventory for Adults scores because of the retrospective nature of this study.

During dry box training, 3D stereoscopic visualization increases the performance related to procedures requiring depth perception especially for novice^25^. In our study, surgeons tended to become LDG operators rather than LTG operators before becoming board certified for laparoscopy (OwoBC ratio of 54% in LDG vs. 28% in LTG): therefore, it is conceivable that the improvement of operative time is greater in LDGs (344 to 269 min, 21.8% reduction) than in LTGs (406 to 351 min, 13.5% reduction) although the operative time baseline under the 2D planar view was shorter in the LDG group.

A previous report by Kanaji *et al*. showed that 3D stereoscopic visualization shortened times for some scenes of lymphadenectomy and for E-J in LTG with D1 + lymphadenectomy (n = 15 in each group), although there was no difference in total operative time^12^. In our study, we only defined the time required for the total dissection procedure, and did not analyse the time required for each part of the lymphadenectomy since defining and identifying the start and end times can be difficult. We found a significant improvement in the time needed for 3D dissection. In their study, they performed D1 + lymphadenectomy that required a shorter operative time than a D2 lymphadenectomy. This could be the reason for the lack of a significant difference in the total operative time between the 3D and 2D groups. In contrast, our study included D2 lymphadenectomies that required more advanced lymph node dissection and longer intracorporeal dissection times, which might have contributed to the significant difference in both total operative and intracorporeal dissection times for the 3D and 2D systems.

A randomized controlled trial (RCT) performed by Lu *et al*., showed that 3D stereoscopic visualization diminished blood loss in laparoscopic gastrectomies^13^. In our study, we found a significant reduction in blood loss with the 3D system in the LDG group, but not in LTG. These results suggest that less bleeding under 3D visualization may also contribute to shorter operative times for LDGs. In their study, however, they did not find a difference in total operative times between their 3D and 2D groups (n = 115 and 113, respectively). Generally speaking, LTG requires a longer operative time than LDG (Table 2). Although this RCT was large, they combined LTG and LDG in their 3D and 2D groups, which could have been the reason that they found no difference in operative times between the two groups.

In conclusion, 3D stereoscopic visualization when compared with 2D, allowed for shorter total operative times in both LTGs and LDGs for gastric cancer. Intracorporeal dissection required shorter times for both LTGs and LDGs under 3D, and the surgeons in this study preferred IKT for intracorporeal anastomosis under 3D.

## Supplementary information

Supplementary Table and Figure

## Data Availability

The datasets in this study are available from the corresponding author on reasonable request.
