# Supplementary material for: Three-dimensional Stereoscopic Visualization Shortens Operative Time in Laparoscopic Gastrectomy for Gastric Cancer
Source: Sci Rep. 2019 Mar 11;9:4108. doi: 10.1038/s41598-019-40269-3 (PMC6411717; doi:10.1038/s41598-019-40269-3)
Supplement: Supplementary file 1 — Supplementary Table and Figure [file 41598_2019_40269_MOESM1_ESM.docx]

**Supplementary information**

**Three-dimensional Stereoscopic Visualization Shortens Operative Time in Laparoscopic Gastrectomy for Gastric Cancer**

Yoshiro Itatani^1*^, Kazutaka Obama^1^, Tatsuto Nishigori^1^, Riki Ganeko^1^, Shigeru Tsunoda^1^, Hisahiro Hosogi^2^, Shigeo Hisamori^1^, Kyoichi Hashimoto^1^, Yoshiharu Sakai^1^

^1^Department of Surgery, Graduate School of Medicine, Kyoto University, Kyoto, 606-8507, Japan

^2^Department of Surgery, Kyoto City Hospital, Kyoto, 604-8845, Japan

*Correspondence should be addressed to Y.I. (email; [itatani@kuhp.kyoto-u.ac.jp](mailto:itatani@kuhp.kyoto-u.ac.jp))

**Supplementary Table S1**

Cases of postoperative complications greater than Clavien-Dindo Grade 2

|  | LTG  3D  (n = 1, 8.3%) | 2D  (n = 4, 31%) | LDG  3D  (n = 2, 6.9%) | 2D  (n = 6, 15%) |
| --- | --- | --- | --- | --- |
| Anastomotic leak |  | 1 (7.7%) |  |  |
| Pancreatic fistula |  | 1 (7.7%) | 2 (6.9%) | 2 (5%) |
| Stasis |  | 1 (7.7%) |  | 1 (2.5%) |
| Postoperative bleeding |  |  |  | 1 (2.5%) |
| Aspiration pneumonia |  | 1 (7.7%) |  | 1 (2.5%) |
| Surgical site infection | 1 (8.3%) |  |  |  |
| Heart failure |  |  |  | 1 (2.5%) |

**Supplementary Figure S1**


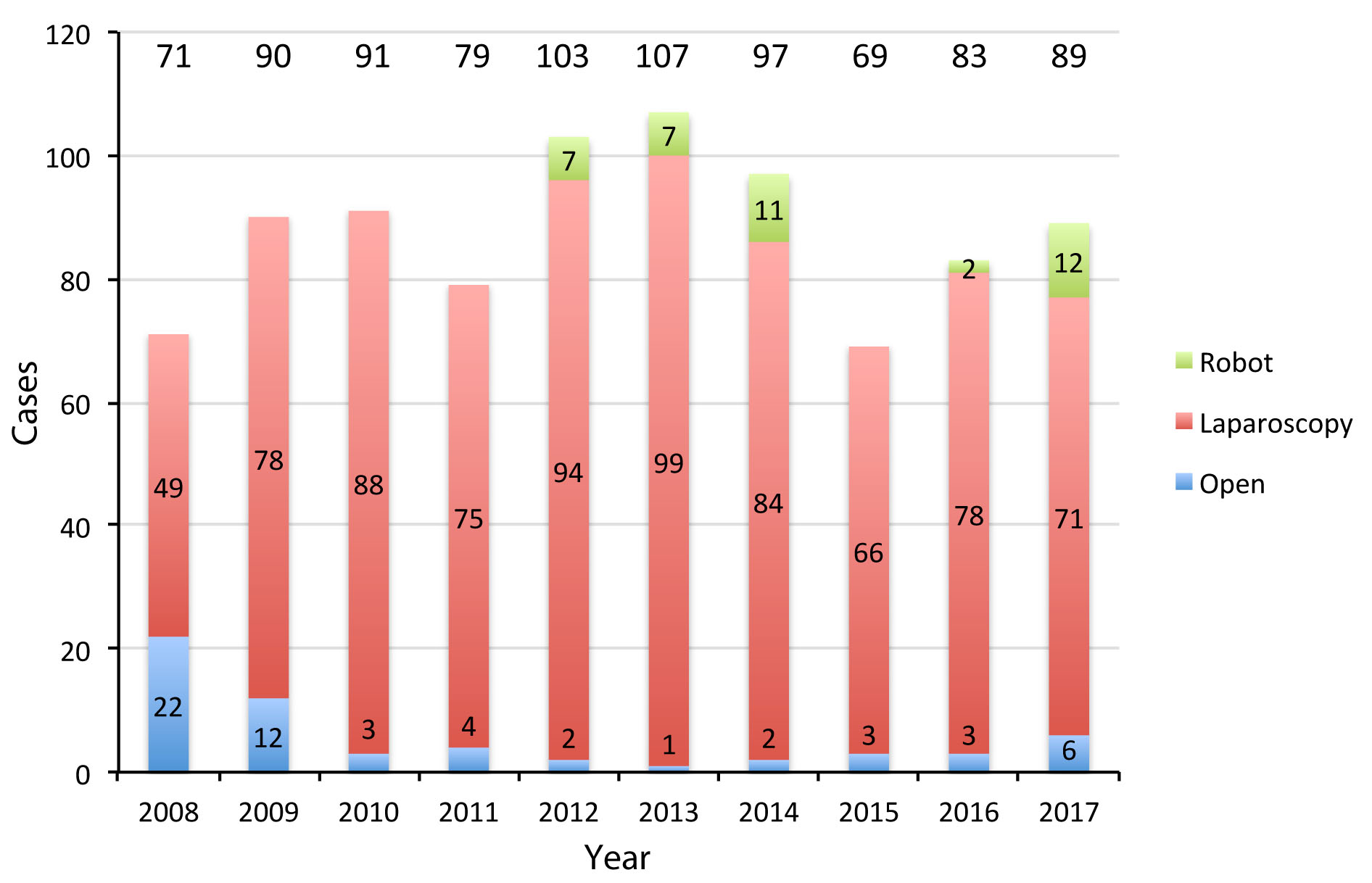


Annual surgical cases for gastric cancer at our institute over a 10-year span. Numbers at the top indicate total number per year.
